# Supplementary material for: MSEDDI: Multi-Scale Embedding for Predicting Drug—Drug Interaction Events
Source: Int J Mol Sci. 2023 Feb 24;24(5):4500. doi: 10.3390/ijms24054500 (PMC10002564; doi:10.3390/ijms24054500)
Supplement: Supplementary file 1 [file ijms-24-04500-s001.zip › ijms-2202863-supplementary.pdf]

Supplementary Materials

MSEDDI: Multi-Scale Embedding for Predicting Drug—Drug Interaction Events

Liyi Yu †, Zhaochun Xu †, Meiling Cheng, Weizhong Lin, Wangren Qiu * and Xuan Xiao *

Department of Computer, School of Information Engineering, Jingdezhen Ceramic University, Jingdezhen 333403, China

* Correspondence: qiuone@163.com (W.Q.); jdzxiaoxuan@163.com (X.X.)

† These authors contributed equally to this work.

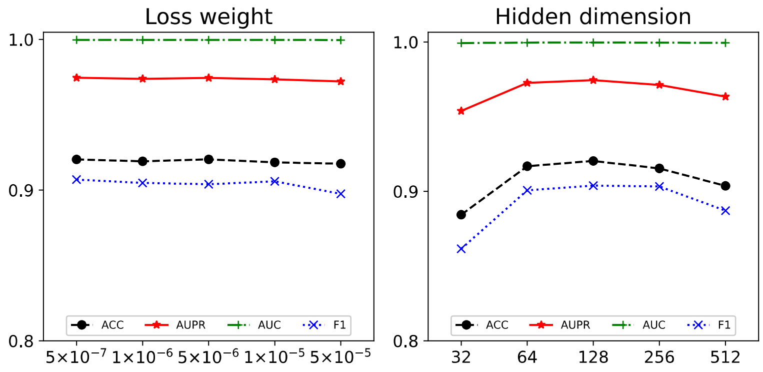

Figure S1. The metric scores under two hyperparameter settings on Database2.

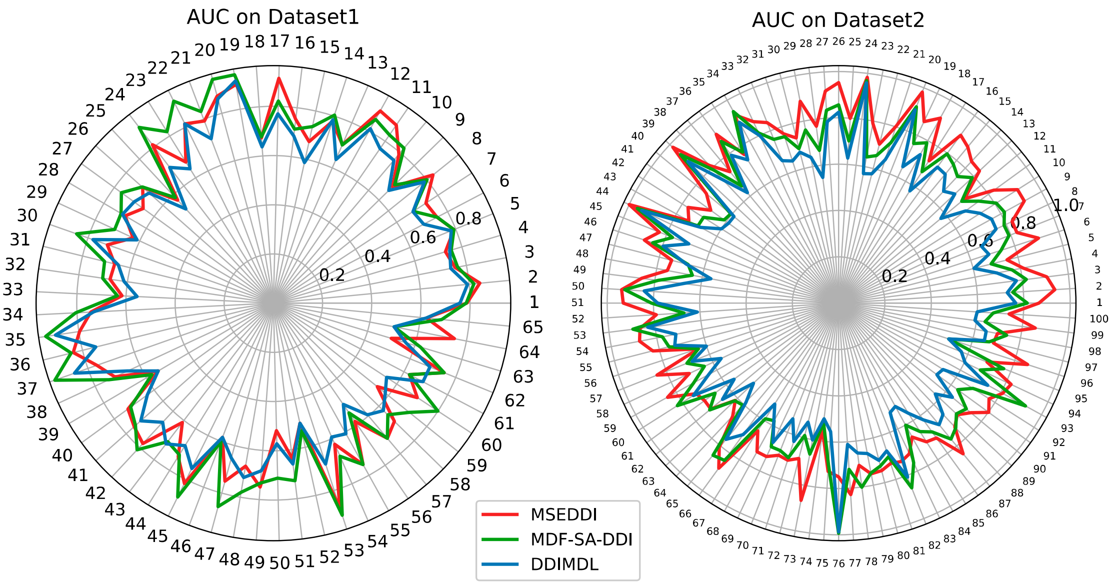

Figure S2. The AUC of partial baselines for each event.

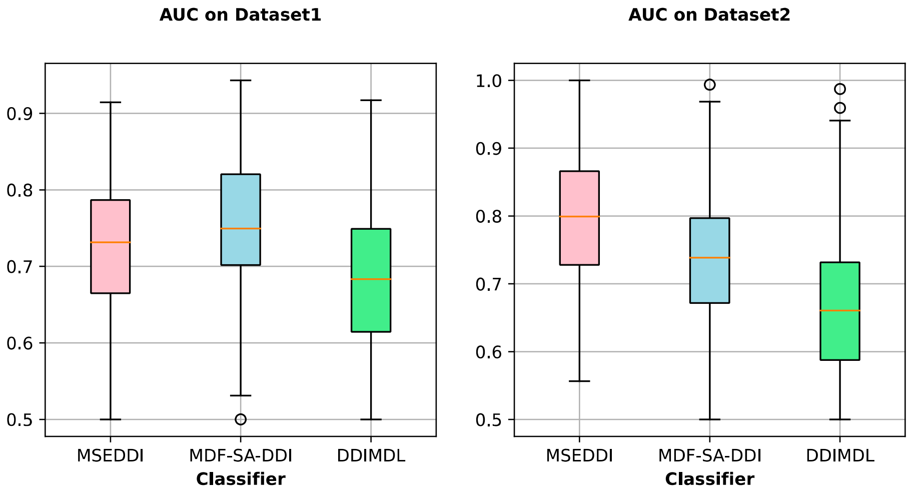

Figure S3. The statistics of AUC for partial baselines in all events.

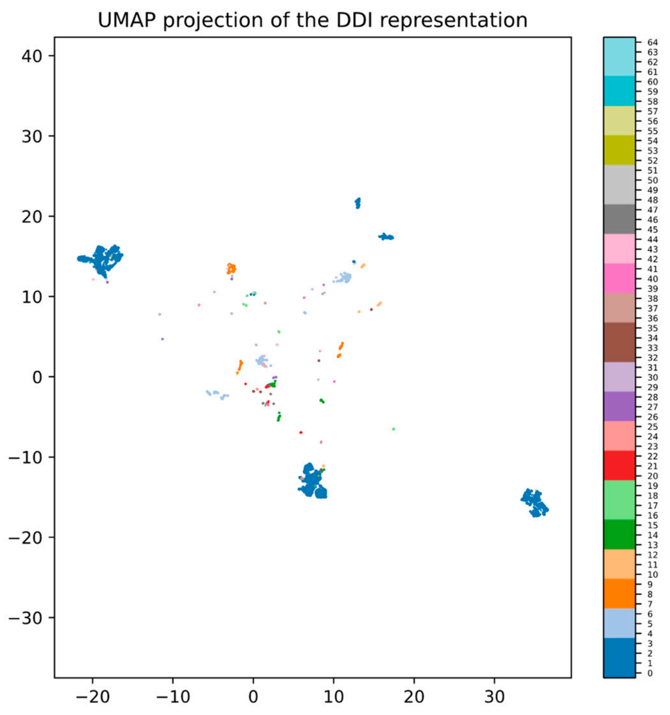

Figure S4. Low-dimensional visualization of drug pair representations through UMAP.

Table S1. The specific hyperparameter settings of MSEDDI on Database1

Hyperparameter		ACC	AUPR	AUC	F1
Weight decay	1e-6	0.8834	0.9513	0.9988	0.7539
	5e-6	0.8845	0.9506	0.9987	0.7345
	1e-5	0.8843	0.9519	0.9988	0.7535
	5e-5	0.8842	0.9519	0.9987	0.7433
	1e-4	0.8894	0.9556	0.9988	0.7244
Dropout	0.05	0.8603	0.9326	0.9982	0.6781
	0.1	0.8815	0.9479	0.9986	0.7485
	0.2	0.8843	0.9519	0.9988	0.7535
	0.3	0.8730	0.9442	0.9986	0.7126
	0.4	0.8384	0.9173	0.9979	0.6459
Learning rate	1e-5	0.7659	0.8392	0.9941	0.3788
	5e-5	0.8671	0.9394	0.9984	0.7027
	1e-4	0.8843	0.9519	0.9988	0.7535
	5e-4	0.8733	0.9442	0.9986	0.7472
	1e-3	0.8689	0.9394	0.9984	0.7181
Hidden dimension	16	0.8269	0.9052	0.9972	0.5253
	32	0.8673	0.9406	0.9984	0.7136
	64	0.8843	0.9519	0.9988	0.7535
	128	0.8753	0.9456	0.9986	0.7434
	256	0.8697	0.9390	0.9983	0.7740
Epoch	250	0.8783	0.9491	0.9987	0.7597
	300	0.8828	0.9501	0.9987	0.7266
	350	0.8843	0.9519	0.9988	0.7535
	400	0.8853	0.9530	0.9988	0.7322
	450	0.8855	0.9525	0.9988	0.7347
Batch size	64	0.8790	0.9486	0.9987	0.7629

	512	0.8843	0.9519	0.9988	0.7535
	1024	0.8793	0.9471	0.9986	0.7373
	1536	0.8672	0.9396	0.9984	0.6840

Table S2. The specific hyperparameter settings of MSEDDI on Database2

Hyperparameter		ACC	AUPR	AUC	F1
Weight decay	5e-7	0.9203	0.9745	0.9996	0.9070
	1e-6	0.9191	0.9739	0.9996	0.9048
	5e-6	0.9204	0.9744	0.9996	0.9039
	1e-5	0.9184	0.9735	0.9996	0.9058
	5e-5	0.9175	0.9721	0.9995	0.8975
Hidden dimension	32	0.8844	0.9539	0.9993	0.8616
	64	0.9168	0.9726	0.9996	0.9007
	128	0.9204	0.9744	0.9996	0.9039
	256	0.9154	0.9713	0.9996	0.9033
	512	0.9037	0.9634	0.9994	0.8871

Table S3. The original AUPR and AUC of partial baselines for each event on Database1

Event	AUPR			AUC		
	MSEDDI	MDF-SA-DDI	DDIMDL	MSEDDI	MDF-SA-DDI	DDIMDL
0	0.7867	0.7844	0.7586	0.7264	0.7233	0.6857
1	0.8436	0.8196	0.7933	0.7722	0.7529	0.7210
2	0.7532	0.7632	0.7350	0.6251	0.6155	0.5883
3	0.7304	0.7546	0.7490	0.5927	0.5884	0.5506
4	0.7925	0.7926	0.7808	0.5735	0.6640	0.5605
5	0.7513	0.7524	0.6905	0.5740	0.5577	0.4323
6	0.7413	0.6808	0.6783	0.5678	0.4341	0.4239
7	0.8322	0.8015	0.7871	0.6823	0.6204	0.5648
8	0.6771	0.6888	0.6558	0.4640	0.4751	0.4379
9	0.7908	0.8243	0.7598	0.6384	0.7426	0.6064
10	0.8797	0.8310	0.7637	0.7342	0.6617	0.5909
11	0.8947	0.8550	0.8118	0.7129	0.6932	0.6602
12	0.7028	0.7011	0.6368	0.4878	0.4608	0.3321
13	0.7762	0.8038	0.7838	0.5934	0.7104	0.5573
14	0.6721	0.7350	0.5851	0.4060	0.4559	0.3007
15	0.7530	0.7124	0.6853	0.5424	0.5444	0.5447
16	0.9144	0.8220	0.7699	0.8240	0.7474	0.6439
17	0.6736	0.6777	0.6367	0.4139	0.4199	0.3364
18	0.9023	0.9424	0.9172	0.7658	0.7764	0.7796
19	0.8714	0.9425	0.8613	0.7429	0.9104	0.7443
20	0.7918	0.8329	0.7146	0.5696	0.7015	0.4464
21	0.8134	0.9155	0.8134	0.6105	0.8467	0.6039
22	0.6750	0.8203	0.6425	0.4795	0.7022	0.4026
23	0.8111	0.8996	0.6945	0.5979	0.7491	0.4956
24	0.5718	0.5858	0.5227	0.2014	0.1717	0.0957
25	0.7106	0.7124	0.6756	0.5116	0.3951	0.4979
26	0.6552	0.7644	0.7004	0.3391	0.6114	0.4621
27	0.7003	0.7188	0.7124	0.4326	0.4471	0.4092
28	0.6290	0.7120	0.6115	0.3506	0.5297	0.2606

29	0.7159	0.8488	0.7784	0.4418	0.6984	0.5382
30	0.6647	0.7074	0.6499	0.3916	0.5518	0.5077
31	0.6716	0.7017	0.6145	0.4474	0.5024	0.4208
32	0.6163	0.6355	0.5555	0.2813	0.3100	0.2835
33	0.7441	0.8058	0.7326	0.5609	0.6035	0.4803
34	0.7968	0.9361	0.8957	0.7226	0.9075	0.8136
35	0.8384	0.8474	0.7443	0.5976	0.7436	0.5833
36	0.7612	0.9430	0.8576	0.6268	0.8372	0.6670
37	0.7114	0.7381	0.6536	0.5232	0.5358	0.3612
38	0.5434	0.5733	0.5434	0.1221	0.2211	0.1158
39	0.7315	0.7177	0.6341	0.5244	0.4925	0.4895
40	0.7567	0.7494	0.7213	0.4667	0.4299	0.5662
41	0.7793	0.8142	0.6616	0.6674	0.7005	0.4516
42	0.6101	0.7081	0.7200	0.2937	0.4490	0.3740
43	0.7345	0.7257	0.6817	0.4504	0.5676	0.4822
44	0.8179	0.8786	0.7498	0.6142	0.7214	0.5503
45	0.5831	0.6021	0.5832	0.1836	0.2826	0.2427
46	0.7498	0.8571	0.6520	0.5116	0.7494	0.3858
47	0.6718	0.7793	0.7187	0.4615	0.5510	0.5106
48	0.7497	0.7332	0.6833	0.4168	0.5251	0.4585
49	0.5178	0.7115	0.5714	0.0598	0.5554	0.2939
50	0.6364	0.7272	0.6591	0.6365	0.5845	0.6592
51	0.4999	0.5312	0.4999	0.0001	0.1147	0.0001
52	0.8750	0.9062	0.6875	0.8750	0.8706	0.5626
53	0.6250	0.6785	0.5938	0.4584	0.3710	0.5939
54	0.7500	0.7500	0.6071	0.5682	0.6389	0.6072
55	0.5833	0.5416	0.5000	0.5834	0.1668	0.0001
56	0.7187	0.7187	0.5937	0.6077	0.4688	0.3439
57	0.6875	0.6500	0.6250	0.4875	0.5251	0.4584
58	0.5417	0.7083	0.5833	0.2918	0.5209	0.3334
59	0.7000	0.8000	0.5500	0.5334	0.8000	0.5501
60	0.6250	0.6250	0.6875	0.6251	0.3750	0.5625
61	0.7500	0.7500	0.6875	0.5357	0.5357	0.6875
62	0.5000	0.6250	0.5625	0.0001	0.3250	0.5626
63	0.7500	0.5000	0.5000	0.3750	0.0000	0.0000
64	0.6250	0.6875	0.6250	0.3251	0.6875	0.4584

Table S4. The original AUPR and AUC of partial baselines for each event on Database2

Event	AUPR			AUC		
	**MSEDDI**	**MDF-SA-DDI**	**DDIMDL**	**MSEDDI**	**MDF-SA-DDI**	**DDIMDL**
0	0.831	0.768	0.7257	0.866	0.8147	0.7649
1	0.8687	0.6256	0.5891	0.9357	0.7541	0.7295
2	0.8153	0.6707	0.6113	0.9062	0.8176	0.7739
3	0.697	0.5963	0.5383	0.8116	0.7636	0.697
4	0.5887	0.4541	0.392	0.7509	0.6802	0.6036
5	0.8185	0.555	0.5135	0.9082	0.7378	0.6826
6	0.737	0.6	0.4936	0.8278	0.7923	0.6647
7	0.7135	0.6127	0.5834	0.8282	0.7894	0.7518
8	0.8216	0.673	0.6226	0.9157	0.8173	0.7539

9	0.8194	0.6558	0.5775	0.9152	0.8062	0.7244
10	0.7272	0.5118	0.4396	0.8297	0.7492	0.6778
11	0.5395	0.4769	0.3433	0.7054	0.6896	0.5735
12	0.7398	0.6697	0.5873	0.8341	0.7916	0.6887
13	0.6845	0.5388	0.4533	0.8389	0.7504	0.663
14	0.7931	0.6482	0.5856	0.8752	0.7771	0.7218
15	0.8006	0.6369	0.5614	0.8964	0.797	0.7259
16	0.5431	0.3327	0.364	0.7278	0.6718	0.5737
17	0.8262	0.6464	0.6049	0.9118	0.7903	0.7318
18	0.7306	0.5764	0.4818	0.8663	0.7618	0.6511
19	0.9432	0.8777	0.8807	0.9809	0.9119	0.8982
20	0.658	0.5529	0.5104	0.8032	0.7283	0.6522
21	0.5313	0.4043	0.3338	0.7085	0.6582	0.5764
22	0.6423	0.3879	0.3738	0.79	0.6428	0.5696
23	0.9707	0.931	0.9318	0.9831	0.9687	0.9594
24	0.6368	0.4946	0.4156	0.7993	0.7019	0.6277
25	0.8566	0.7437	0.6826	0.9526	0.8567	0.8248
26	0.8341	0.75	0.7179	0.9175	0.8347	0.7851
27	0.6034	0.4358	0.2714	0.7695	0.6645	0.5469
28	0.794	0.6011	0.5001	0.8868	0.7615	0.6411
29	0.6407	0.5168	0.5377	0.7367	0.6885	0.6732
30	0.6256	0.5448	0.4793	0.7684	0.7561	0.6456
31	0.7224	0.5635	0.4884	0.8387	0.7602	0.6612
32	0.7818	0.6716	0.6492	0.866	0.8119	0.8
33	0.8857	0.9162	0.8537	0.9268	0.9439	0.8911
34	0.6181	0.63	0.6026	0.7601	0.7724	0.733
35	0.7877	0.7774	0.709	0.9006	0.8627	0.7897
36	0.634	0.4776	0.4347	0.7366	0.6573	0.619
37	0.704	0.5841	0.5473	0.8154	0.7881	0.7318
38	0.9437	0.8817	0.8091	0.9831	0.9373	0.8937
39	0.4746	0.3651	0.3126	0.7409	0.6053	0.5864
40	0.4982	0.3684	0.3845	0.6824	0.6112	0.5876
41	0.5939	0.4343	0.3497	0.7707	0.6912	0.5997
42	0.645	0.4161	0.4199	0.7385	0.6932	0.6795
43	0.9859	0.9578	0.9359	1	0.9618	0.9408
44	0.7348	0.6607	0.5502	0.8327	0.7719	0.6957
45	0.7662	0.7447	0.6907	0.8823	0.8448	0.797
46	0.639	0.5344	0.5032	0.7442	0.6708	0.6812
47	0.6066	0.3829	0.308	0.7536	0.6437	0.5617
48	0.7316	0.6177	0.475	0.8578	0.7862	0.6771
49	0.899	0.876	0.7397	0.939	0.9258	0.8414
50	0.8435	0.679	0.6219	0.931	0.8085	0.7816
51	0.69	0.5185	0.5211	0.789	0.6689	0.612
52	0.8075	0.8418	0.7788	0.865	0.8961	0.8316
53	0.7651	0.6594	0.6386	0.8759	0.7755	0.7155
54	0.716	0.5515	0.5552	0.8289	0.7569	0.7306
55	0.4347	0.4631	0.4897	0.6372	0.6374	0.6
56	0.7239	0.5255	0.521	0.8439	0.705	0.6808
57	0.5103	0.471	0.3545	0.7133	0.6411	0.561
58	0.6651	0.5925	0.4565	0.8433	0.715	0.6411
59	0.5975	0.7093	0.5989	0.7285	0.8251	0.7438

60	0.5415	0.5197	0.383	0.7155	0.6603	0.5582
61	0.555	0.6064	0.5101	0.7165	0.7233	0.6516
62	0.3357	0.4899	0.1926	0.6128	0.6146	0.5048
63	0.4432	0.3226	0.3321	0.6684	0.6042	0.5845
64	0.7702	0.7025	0.6597	0.8084	0.8518	0.7629
65	0.8131	0.7259	0.6291	0.8807	0.8387	0.7016
66	0.362	0.4223	0.251	0.6524	0.5911	0.5155
67	0.6656	0.3825	0.4207	0.7321	0.579	0.5812
68	0.6324	0.3591	0.2973	0.7323	0.6846	0.665
69	0.6138	0.5709	0.4051	0.706	0.6849	0.5365
70	0.6308	0.5712	0.5069	0.7131	0.7011	0.6277
71	0.4837	0.3517	0.2592	0.6923	0.5896	0.5299
72	0.7941	0.4715	0.4116	0.8668	0.6629	0.6011
73	0.2006	0.1185	0.1032	0.5565	0.5211	0.503
74	0.4953	0.607	0.4648	0.7279	0.7006	0.5556
75	0.7036	0.9636	0.9403	0.75	0.9937	0.9873
76	0.781	0.6333	0.5557	0.8277	0.7187	0.6111
77	0.5007	0.7286	0.3398	0.7058	0.8007	0.6521
78	0.7429	0.563	0.3976	0.75	0.7464	0.6607
79	0.7034	0.7177	0.6838	0.7461	0.7254	0.7007
80	0.5823	0.5262	0.4874	0.7276	0.6969	0.6704
81	0.6851	0.7621	0.6851	0.7351	0.8577	0.8077
82	0.6132	0.7282	0.3972	0.7187	0.761	0.5221
83	0.4122	0.3958	0.1755	0.6591	0.6009	0.5088
84	0.489	0.4406	0.3469	0.7089	0.6567	0.5634
85	0.6578	0.6474	0.579	0.7192	0.6865	0.623
86	0.7628	0.6712	0.5166	0.8437	0.7615	0.6807
87	0.6924	0.5887	0.4925	0.7333	0.7386	0.6591
88	0.4828	0.4332	0.363	0.7031	0.6975	0.5726
89	0.7095	0.5677	0.4047	0.7417	0.7155	0.6379
90	0.7147	0.4721	0.5247	0.8016	0.6352	0.5246
91	0.6503	0.6096	0.434	0.77	0.6864	0.5339
92	0.693	0.8516	0.5245	0.8088	0.9216	0.7353
93	0.7073	0.4923	0.4651	0.8235	0.6765	0.6029
94	0.4779	0.6508	0.2921	0.6887	0.7674	0.5698
95	0.8358	0.0003	0.5003	0.8586	0.5	0.5
96	0.5972	0.3337	0.3751	0.6902	0.6722	0.65
97	0.5489	0.6806	0.6006	0.6988	0.7394	0.6489
98	0.7038	0.6158	0.4199	0.8562	0.6375	0.5937
99	0.6506	0.5063	0.4637	0.7375	0.6687	0.6

Table S5. The performance of MSEDDI variants on Dataset1

Method	Task1				Task2			
	ACC	AUPR	AUC	F1	ACC	AUPR	AUC	F1
MSEDDI_NC	0.6281	0.6676	**0.9842**	0.4218	0.4192	0.3925	**0.9620**	0.1310
MSEDDI_SC	0.5152	0.5118	0.9682	0.2661	0.2843	0.2203	0.9256	0.0540
MSEDDI_GC	0.5008	0.4983	0.9630	0.2039	0.3115	0.2563	0.9221	0.0734
MSEDDI_NO_ADD	0.6428	0.6765	0.9819	0.4765	0.4262	0.3881	0.9529	0.1546
MSEDDI_NO_CONV	0.6328	0.6670	0.9816	0.4732	0.4089	0.3728	0.9528	0.1585
MSEDDI_NO_ATTEN	0.6112	0.6347	0.9805	0.3711	0.3993	0.3432	0.9488	0.0908
**MSEDDI**	**0.6517**	**0.6810**	0.9823	**0.4771**	**0.4451**	**0.3999**	0.9543	**0.1691**

The best results are highlighted in boldface.

Table S6. The performance of MSEDDI variants on Dataset2

Method	Task1				Task2			
	ACC	AUPR	AUC	F1	ACC	AUPR	AUC	F1
MSEDDI_NC	0.7245	0.7873	0.9938	0.5778	0.6062	0.6369	0.9863	0.2869
MSEDDI_SC	0.5442	0.5573	0.9831	0.3936	0.3082	0.2314	0.9391	0.0933
MSEDDI_GC	0.5383	0.5451	0.9817	0.3939	0.3556	0.2966	0.9530	0.1577
MSEDDI_NO_ADD	**0.7698**	**0.8317**	0.9947	0.6419	**0.6316**	**0.6632**	0.9865	0.3058
MSEDDI_NO_CONV	0.7659	0.8288	**0.9948**	0.6350	0.6248	0.6557	**0.9865**	0.3011
MSEDDI_NO_ATTEN	0.7512	0.8130	0.9943	0.6138	0.6046	0.6276	0.9848	0.2780
**MSEDDI**	0.7697	0.8315	0.9947	**0.6486**	0.6309	0.6596	0.9863	**0.3111**

The best results are highlighted in boldface.

Table S7. MSEDDI predictions for DDI events unknown in Dataset

Drug1	Drug2	Event	Label	
Lapatinib	Mestranol	The metabolism decrease	Event#0	
Nabiximols	Losartan	The metabolism decrease	Event#0	reverse
Brivaracetam	Midostaurin	The metabolism decrease	Event#0	reverse
Midostaurin	Methoxyflurane	The metabolism decrease	Event#0	reverse
Nateglinide	Mexiletine	The metabolism decrease	Event#0	
Brivaracetam	Imatinib	The metabolism decrease	Event#0	reverse
Brivaracetam	Lobeglitazone	The metabolism decrease	Event#0	reverse
Lobeglitazone	Ketamine	The metabolism decrease	Event#0	reverse
Enasidenib	Lorcaserin	The metabolism decrease	Event#0	reverse
Arformoterol	Cholecalciferol	The metabolism decrease	Event#0	reverse
Brivaracetam	Bortezomib	The metabolism decrease	Event#0	reverse
Donepezil	Cholecalciferol	The metabolism decrease	Event#0	reverse

Cholecalciferol	Cevimeline	The metabolism decrease	Event#0	reverse
Encainide	Cholecalciferol	The metabolism decrease	Event#0	reverse
Ethylmorphine	Cholecalciferol	The metabolism decrease	Event#0	reverse
Cholecalciferol	Antipyrine	The metabolism decrease	Event#0	reverse
Cholecalciferol	Methoxyflurane	The metabolism decrease	Event#0	reverse
Enasidenib	Cholecalciferol	The metabolism decrease	Event#0	reverse
Mesoridazine	Fospropofol	The risk or severity of adverse effects increase	Event#1	reverse
Brompheniramine	Fospropofol	The risk or severity of adverse effects increase	Event#1	reverse
Meprobamate	Dextropropoxyphene	The risk or severity of adverse effects increase	Event#1	reverse
Mesoridazine	Meprobamate	The risk or severity of adverse effects increase	Event#1	reverse
Hydromorphone	Meclizine	The risk or severity of adverse effects increase	Event#1	reverse
Meprobamate	Lurasidone	The risk or severity of adverse effects increase	Event#1	reverse
Hydromorphone	Ketazolam	The risk or severity of adverse effects increase	Event#1	reverse
Fospropofol	Almotriptan	The risk or severity of adverse effects increase	Event#1	
Almotriptan	Fospropofol	The risk or severity of adverse effects increase	Event#1	
Meclizine	Ketazolam	The risk or severity of adverse effects increase	Event#1	reverse
Meprobamate	Dantrolene	The risk or severity of adverse effects increase	Event#1	reverse
Meprobamate	Ezogabine	The risk or severity of adverse effects increase	Event#1	reverse
Methsuximide	Meprobamate	The risk or severity of adverse effects increase	Event#1	reverse

Morphine	Ketazolam	The risk or severity of adverse effects increase	Event#1	reverse
Meprobamate	Meclizine	The risk or severity of adverse effects increase	Event#1	reverse
Meprobamate	Glutethimide	The risk or severity of adverse effects increase	Event#1	reverse
Meprobamate	Fospropofol	The risk or severity of adverse effects increase	Event#1	reverse
Meprobamate	Melperone	The risk or severity of adverse effects increase	Event#1	reverse
Meprobamate	Ketazolam	The risk or severity of adverse effects increase	Event#1	reverse
Carbamazepine	Naproxen	The metabolism increase	Event#8	reverse
Fosphenytoin	Fenofibrate	The metabolism increase	Event#8	reverse
Carbamazepine	Antipyrine	The metabolism increase	Event#8	reverse
Acetaminophen	Amobarbital	The metabolism increase	Event#8	reverse
Clofibrate	Carbamazepine	The metabolism increase	Event#8	reverse
Fenofibrate	Carbamazepine	The metabolism increase	Event#8	reverse
Iloperidone	Domperidone	The risk or severity of QTc prolongation increase	Event#24	reverse
Cisapride	Anagrelide	The risk or severity of QTc prolongation increase	Event#24	reverse
Citalopram	Asenapine	The risk or severity of QTc prolongation increase	Event#24	reverse
Iloperidone	Disopyramide	The risk or severity of QTc prolongation increase	Event#24	reverse
Domperidone	Citalopram	The risk or severity of QTc prolongation increase	Event#24	reverse
Cisapride	Asenapine	The risk or severity of QTc	Event#24	reverse

		prolongation increase		
Flupentixol	Cisapride	The risk or severity of QTc prolongation increase	Event#24	reverse
Fluoxetine	Disopyramide	The risk or severity of QTc prolongation increase	Event#24	reverse
Escitalopram	Disopyramide	The risk or severity of QTc prolongation increase	Event#24	reverse
Disopyramide	Citalopram	The risk or severity of QTc prolongation increase	Event#24	reverse
Domperidone	Asenapine	The risk or severity of QTc prolongation increase	Event#24	reverse
Flupentixol	Eliglustat	The risk or severity of QTc prolongation increase	Event#24	reverse
Citalopram	Flupentixol	The risk or severity of QTc prolongation increase	Event#24	reverse
Etonogestrel	Acetohexamide	The therapeutic efficacy decrease	Event#4	reverse
Ethinylestradiol	Glimepiride	The therapeutic efficacy decrease	Event#4	reverse
Levonorgestrel	Glyburide	The therapeutic efficacy decrease	Event#4	reverse
Donepezil	Cyclopentolate	The therapeutic efficacy decrease	Event#4	reverse
Mestranol	Glimepiride	The therapeutic efficacy decrease	Event#4	reverse
Levonorgestrel	Glipizide	The therapeutic efficacy decrease	Event#4	reverse
Estradiol	Glimepiride	The therapeutic efficacy decrease	Event#4	reverse
Gestodene	Glipizide	The therapeutic efficacy decrease	Event#4	
Gestodene	Acetohexamide	The therapeutic efficacy decrease	Event#4	
Gestodene	Chlorpropamide	The therapeutic efficacy decrease	Event#4	
Etonogestrel	Gliclazide	The therapeutic efficacy decrease	Event#4	reverse

Mitiglinide	Levonorgestrel	The therapeutic efficacy decrease	Event#4	reverse
Levonorgestrel	Glimepiride	The therapeutic efficacy decrease	Event#4	reverse
Gestodene	Glyburide	The therapeutic efficacy decrease	Event#4	
Gestodene	Gliclazide	The therapeutic efficacy decrease	Event#4	
Levonorgestrel	Acetohexamide	The therapeutic efficacy decrease	Event#4	reverse
Gestodene	Glimepiride	The therapeutic efficacy decrease	Event#4	
Levonorgestrel	Gliclazide	The therapeutic efficacy decrease	Event#4	reverse

The "reverse" means the corresponding reverse-order drug-drug pair (DDI) exists in the training set.

Table S8. All DDI event profiles for Database1

Event	Label
The metabolism decrease	0
The risk or severity of adverse effects increase	1
The serum concentration increase	2
The serum concentration decrease	3
The therapeutic efficacy decrease	4
the central nervous system depressant (CNS depressant) activities increase	5
the QTc - prolonging activities increase	6
the hypotensive activities increase	7
The metabolism increase	8
the antihypertensive activities decrease	9
the hypoglycemic activities increase	10
the anticoagulant activities increase	11
The serum concentration of the active metabolites increase	12
the bradycardic activities increase	13
the serotonergic activities increase	14
The therapeutic efficacy increase	15
the hypokalemic activities increase	16
the orthostatic hypotensive activities increase	17
the cardiotoxic activities decrease	18
the excretion rate which could result in a higher serum level decrease	19
the atrioventricular blocking (AV block) activities increase	20
the sedative activities decrease	21
the tachycardic activities increase	22
the hypertensive and vasoconstricting activities increase	23
The risk or severity of QTc prolongation increase	24
the antihypertensive activities increase	25
the arrhythmogenic activities increase	26
the cardiotoxic activities increase	27

The risk or severity of hypotension increase	28
the bronchodilatory activities decrease	29
the hyperkalemic activities increase	30
the nephrotoxic activities increase	31
the hypertensive activities increase	32
the vasoconstricting activities increase	33
the neuroexcitatory activities increase	34
the anticoagulant activities decrease	35
the fluid increase	36
the antiplatelet activities increase	37
The serum concentration of the active metabolites reduce	38
the immunosuppressive activities increase	39
The risk or severity of bleeding increase	40
the stimulatory activities decrease	41
the vasoconstricting activities decrease	42
The risk or severity of myelosuppression increase	43
the thrombogenic activities increase	44
the anticholinergic activities increase	45
the analgesic activities increase	46
an increase in the absorption resulting in an increased serum concentration and potentially a worsening of adverse effects cause	47
The risk or severity of sedation and somnolence increase	48
The risk or severity of rhabdomyolysis increase	49
The risk or severity of hyperkalemia increase	50
the hepatotoxic activities increase	51
the respiratory depressant activities increase	52
the myopathic rhabdomyolysis activities increase	53
the vasopressor activities increase	54
a decrease in the absorption resulting in a reduced serum concentration and potentially a decrease in efficacy cause	55
the hyponatremic activities increase	56
the excretion rate which could result in a lower serum level and potentially a reduction in efficacy increase	57
The risk or severity of myopathy and rhabdomyolysis increase	58
the neuromuscular blocking activities decrease	59
The risk of a hypersensitivity reaction increase	60
the hypocalcemic activities increase	61
the vasodilatory activities increase	62
the myelosuppressive activities increase	63
the hyperglycemic activities increase	64

Table S9. All DDI event profiles for Database2

Event	Label
The metabolism decrease	0

the excretion rate which could result in a higher serum level decrease	1
The risk or severity of adverse effects increase	2
The metabolism increase	3
The serum concentration increase	4
The risk or severity of QTc prolongation increase	5
The therapeutic efficacy decrease	6
the central nervous system depressant (CNS depressant) activities increase	7
the antihypertensive activities decrease	8
The risk or severity of hypertension increase	9
the excretion rate which could result in a lower serum level and potentially a reduction in efficacy increase	10
The serum concentration decrease	11
The therapeutic efficacy increase	12
the hypotensive activities increase	13
the arrhythmogenic activities increase	14
The risk or severity of hyperkalemia increase	15
The excretion decrease	16
The risk or severity of hypoglycemia increase	17
The risk or severity of bleeding increase	18
The risk or severity of hyperglycemia increase	19
the bradycardic activities increase	20
The risk or severity of serotonin syndrome increase	21
The risk or severity of myopathy rhabdomyolysis and myoglobinuria increase	22
The risk or severity of gastrointestinal irritation increase	23
The risk or severity of hypotension increase	24
The risk or severity of bradycardia increase	25
The risk or severity of bleeding and hemorrhage increase	26
the orthostatic hypotensive activities increase	27
the hypoglycemic activities increase	28
The risk or severity of CNS depression increase	29
a decrease in the absorption resulting in a reduced serum concentration and potentially a decrease in efficacy cause	30
The risk or severity of Tachycardia increase	31
The risk or severity of gastrointestinal bleeding increase	32
The risk or severity of renal failure hyperkalemia and hypertension increase	33
the antihypertensive activities increase	34
The risk or severity of hypokalemia increase	35
the immunosuppressive activities increase	36
the neuromuscular blocking activities increase	37
the neuroexcitatory activities increase	38
the orthostatic hypotensive hypotensive and antihypertensive activities increase	39
the QTcprolonging activities increase	40
the serotonergic activities increase	41
The risk or severity of sedation increase	42

The risk or severity of tendinopathy increase	43
the anticoagulant activities increase	44
the hypertensive and vasoconstricting activities increase	45
the sedative activities increase	46
the hypertensive activities increase	47
the vasoconstricting activities increase	48
The protein binding decrease	49
the anticoagulant activities decrease	50
the neurotoxic activities increase	51
The risk or severity of edema formation increase	52
the hypoglycemic activities decrease	53
the vasodilatory activities increase	54
The risk or severity of methemoglobinemia increase	55
The risk or severity of hemorrhage increase	56
The risk or severity of nephrotoxicity increase	57
the sedative and stimulatory activities decrease	58
the nephrotoxic activities increase	59
the anticholinergic activities increase	60
The risk or severity of hypotension dyspepsia and headache increase	61
the hepatotoxic activities increase	62
The risk or severity of seizure increase	63
the tachycardic activities increase	64
the bronchodilatory activities decrease	65
the antiplatelet activities increase	66
The risk or severity of myelosuppression increase	67
The risk or severity of hypotension and orthostatic hypotension increase	68
The risk or severity of Cardiac Arrhythmia increase	69
the hyperkalemic activities increase	70
The risk or severity of orthostatic hypotension and syncope increase	71
the analgesic activities increase	72
The risk or severity of liver damage increase	73
The risk or severity of extrapyramidal symptoms increase	74
The risk or severity of electrolyte imbalance increase	75
the thrombogenic activities increase	76
The risk or severity of sedation and somnolence increase	77
The risk or severity of myopathy and weakness increase	78
The risk or severity of renal failure and hypertension increase	79
The risk or severity of hypertension decrease	80
The risk or severity of neutropenia and thrombocytopenia increase	81
The risk or severity of Tachycardia and drowsiness increase	82
The risk or severity of neutropenia increase	83
the stimulatory activities decrease	84
The risk or severity of renal failure increase	85
The risk or severity of fluid retention increase	86

The risk or severity of hyponatremia increase	87
the vasopressor activities increase	88
The risk or severity of QTc prolongation decrease	89
The risk or severity of hypotension and CNS depression increase	90
an increase in the absorption resulting in an increased serum concentration and potentially a worsening of adverse effects cause	91
The risk or severity of renal failure hypotension and hyperkalemia increase	92
The risk or severity of QTc prolongation and hypotension increase	93
The risk or severity of angioedema increase	94
The serum concentration of the active metabolites increase	95
The absorption decrease	96
the hypokalemic activities increase	97
the neuromuscular blocking activities decrease	98
The risk or severity of hyperthermia and oligohydrosis increase	99
